# The Draft Genome Sequence of Pseudomonas frederiksbergensis Strain 11-D3 Reveals Its Ability To Mobilize Phosphorus

**DOI:** 10.1128/MRA.01321-18

**Published:** 2019-03-14

**Authors:** Bang-Xiao Zheng, Huang-Kai Zhang, Yu-Sen Zhang, Kai Ding

**Affiliations:** a Key Laboratory of Urban Environment and Health, Institute of Urban Environment, Chinese Academy of Sciences, Xiamen, People’s Republic of China; b Faculty of Biological and Environmental Sciences, Ecosystems and Environment Research Programme, University of Helsinki, Lahit, Finland; c Xiamen Anjie Medical Data Technology Co. Ltd., Xiamen, People’s Republic of China; University of California, Riverside

## Abstract

Pseudomonas frederiksbergensis strain 11-D3 is a Gram-negative, nonmotile, aerobic bacterium isolated from a managed maize field in North China. This strain displays high efficiency for solubilization of inorganic phosphorus (P). We present the draft genome sequence of strain 11-D3 with P cycling genes which indicate the presence of a probable mechanism for P mobilization.

## Abstract (precis)

Pseudomonas frederiksbergensis strain 11-D3 is a Gram-negative, nonmotile, aerobic bacterium isolated from a managed maize field in North China. This strain displays high efficiency for solubilization of inorganic phosphorus (P).

## ANNOUNCEMENT

Species of the genus *Pseudomonas* are commonly considered important decomposers of organic compounds in soil and water (1). Certain *Pseudomonas* strains have biotechnological importance due to their significant plant growth promotion effects, including control of fungal disease and mobilization of phosphorus (P) (2). Pseudomonas frederiksbergensis was first reported in 2000 (3) and then was proved to have the ability to oxidate organic sulfides (4). Strain 11-D3 was isolated from a managed maize field in Hailun, Heilongjiang Province, China (47°26′N, 126°38′E), by using a rapid isolation method (5). This bacterium is of interest because similar strains have shown the ability to solubilize P from diverse inorganic sources, including calcium, aluminum, and ferric phosphate (6).

Strain 11-D3 is an aerobic bacterium which survives best at 30°C and is viable in tested pH ranges from 5.5 to 8.0 (5). To taxonomically identify the affiliation of strain 11-D3, the 16S rRNA sequence (NCBI accession number KU647205) was amplified with the bacterial universal primers 27F and 1492R (7), obtained by Sanger sequencing (Invitrogen, Shanghai, China), and submitted to EzBioCloud for alignment with the type strain database (8). This sequence contains 1,444 bp (99.0% of the full-length 16S rRNA), and the highest sequence identity match of 98.68% was with P. frederiksbergensis strain JAJ28^T^. Hence, we propose the affiliation of strain 11-D3 with P. frederiksbergensis.

Strain 11-D3 was cultured on Pikovskaya (PVK) medium (9) at 30°C for 24 h, and a single colony was picked for culture in liquid PVK medium for another 24-h cultivation period. The strain cells were collected by centrifugation at 5,000 × *g* for 15 min, and the genomic DNA was extracted by using a genomic DNA extraction kit for bacteria (Bioteck, Beijing, China) according to the manufacturer’s instructions. The draft genome was sequenced by using an Illumina HiSeq 2000 platform (Majorbio, Shanghai, China). Trim Galore v0.3.2 (https://www.bioinformatics.babraham.ac.uk/projects/trim_galore/) was used to remove adapters and low-quality bases and reads. A 1,139-Mb sequence, with 1,223 Mb of total reads, was assembled by using the Short Oligonucleotide Analysis Package (SOAPdenovo v2.01) with all parameters set to default (10). The submitted genome sequence was autoannotated by using the NCBI Prokaryotic Genome Annotation Pipeline with default settings (11). The draft genome assembly of strain 11-D3 contains 35 contigs in 35 scaffolds, and the total genome size is 6,524,485 bp. The G+C content is 59.44%. Genome annotation produced 6,211 genes, of which 5,839 (94.01%) are protein-coding genes. Two gene clusters (*pqqEDCBA* and *pqqFECBA*) involved in pyrroloquinoline quinone (PQQ) biosynthesis were identified from the genome of strain 11-D3, which have been proven to be related to its capacity to solubilize inorganic phosphate (12). According to previous studies, the *pqq* operon is a key regulation factor for the expression of gluconic acid and 2-keto-d-gluconic acid, which are important for high-efficiency inorganic P solubilization in the genus *Pseudomonas* (13–15). Two alkaline phosphatase genes (one belongs to phosphodiest superfamily and the other is *phoD*) were also found. Three phosphatase genes, *phoA*, *phoX*, and *phoD*, have been reported to be widely distributed in diverse habitats (16), and *phoD* is commonly considered a gene marker for bacteria that mineralize soil P (17, 18). The findings reported here may indicate the high efficiency of strain 11-D3 for inorganic P solubilization.

### Data availability.

This whole-genome shotgun project was deposited at DDBJ/ENA/GenBank under the accession number PUIN00000000. The version described in this paper is version PUIN01000000. The raw sequences were deposited in the Sequence Read Archive (SRX3744610).
